# Efficacy and Safety of Laser Balloon Versus Irrigated Radiofrequency Ablation as Initial Therapies for Atrial Fibrillation: A Meta-Analysis

**DOI:** 10.31083/j.rcm2506205

**Published:** 2024-06-03

**Authors:** Fujiang Chen, Donglin Guo, Tiantian Zheng, Yangyang Gu, Xinbin Zhou, Yuangang Qiu, Shuwei Huang, Wenyi Ye

**Affiliations:** ^1^Department of Anesthesiology, Shanxi Provincial People's Hospital, 030012 Taiyuan, Shanxi, China; ^2^Department of Clinical Laboratory, Shanxi Provincial People's Hospital, 030012 Taiyuan, Shanxi, China; ^3^The First College of Clinical Medicine, Zhejiang Chinese Medical University, 310053 Hangzhou, Zhejiang, China; ^4^The First Affiliated Hospital of Zhejiang Chinese Medical University (Zhejiang Provincial Hospital of Chinese Medicine), 310006 Hangzhou, Zhejiang, China

**Keywords:** atrial fibrillation, catheter ablation, laser balloon ablation, radiofrequency ablation, meta-analysis

## Abstract

**Background:**

Catheter ablation (CA) is an effective therapy for atrial fibrillation (AF) and, although radiofrequency ablation (RFA) is the standard treatment for pulmonary vein isolation (PVI), it is complex and time-consuming. Laser balloon ablation (LBA) has been introduced to simplify the conventional RFA; however, results of studies comparing LBA and RFA remain controversial. As such, this investigation aimed to comprehensively evaluate the efficacy and safety of LBA versus RFA.

**Methods:**

The PubMed, Embase, Cochrane Library, and ClinicalTrials.gov databases were searched for relevant studies. The primary endpoints were the freedom from atrial tachyarrhythmia (ATA) and procedure-related complications.

**Results:**

Twelve studies including 1274 subjects were included. LBA and RFA yielded similar rates of freedom from ATA (72.5% vs. 68.7%, odds ratio [OR] = 1.26, 95% confidence interval [CI] 1.0–1.7, *p* = 0.11) and procedure-related complications (7.7% vs. 6.5%, OR = 1.17, 95% CI 0.72–1.90,* p* = 0.536). LBA with the second- and third-generation laser balloons (LB2/3) yielded remarkably higher rates of freedom from ATA than RFA using contact-force technology (RFA-CF) (OR = 1.91, *p *= 0.013). Significantly lower pulmonary vein (PV) reconnection rates (OR = 0.51, *p* = 0.021), but higher phrenic nerve palsy (PNP) rates (OR = 3.42, *p* = 0.023) were observed in the LBA group. LBA had comparable procedure (weighted mean difference [WMD] = 8.43 min, *p* = 0.337) and fluoroscopy times (WMD = 3.09 min, *p* = 0.174), but a longer ablation time (WMD = 12.57 min, *p* = 0.00) than those for RFA.

**Conclusions:**

LBA and RFA treatments were comparable in terms of freedom from ATA and postprocedural complications in patients with AF. Compared with RFA, LBA was associated with significantly lower PV reconnection rates, but a higher incidence of PNP and longer ablation time.

## 1. Introduction

Atrial fibrillation (AF) is the most common arrhythmia, and increases the risk for stroke, heart failure, and mortality [1]. Catheter ablation (CA) is an effective therapy for restoring and maintaining sinus rhythm in patients with AF, with pulmonary vein isolation (PVI) being the cornerstone of various CA procedures [2].

Irrigated radiofrequency ablation (RFA) is the standard therapy for PVI; however, this conventional strategy is time-consuming and technically complex [3]. Recently, balloon-based CA technologies, including the laser balloon ablation (LBA), have been applied in clinical practice to simplify RFA and overcome its complexity [4]. In addition, several newly developed technologies, such as LBA with more compliant second- and third-generation laser balloons (LB2/3) and RFA with contact-force technology (RFA-CF), have also been introduced and are widely used [5]. Previous studies have directly compared LBA and RFA; however, results remain controversial. High-quality studies comparing newly updated techniques for LB2/3 versus RFA-CF are lacking. As such, this meta-analysis aimed to comprehensively assess the safety, efficacy, and procedural characteristics of LBA and RFA.

## 2. Materials and Methods

### 2.1 Literature Search and Selection Criteria

The PubMed/Medline, Embase, Cochrane Library, and ClinicalTrials.gov databases were searched for relevant studies published up to October 2023. The following keywords and their variants were used: “atrial fibrillation”, “laser balloon”, “radiofrequency ablation”. The reference lists of all retrieved studies were also searched for other potentially eligible studies. Clinical trials that fulfilled the following criteria were included: articles published in English with full text; direct comparison of LBA and RFA treatments as initial therapies for patients with AF; and adequate data regarding to the primary and secondary outcomes.

### 2.2 Data Extraction and Quality Assessment

Two authors (FJC and DLG) extracted the data of interest and independently assessed quality. Discrepancies were resolved through consensus discussion with a third author (XBZ). The following data were extracted from the included studies: publication year, sample size, baseline characteristics of participants, AF type, LBA and RFA protocols, and other relevant outcomes. The Cochrane Collaboration tool was used to evaluate the quality of the randomized controlled trials (RCTs) [6] and the Risk of Bias In Non-randomized Studies of Interventions (i.e., “ROBINS-I”) [7] for the non-randomized trials.

### 2.3 Primary and Secondary Outcomes 

Primary outcomes included freedom from atrial tachyarrhythmia (ATA), defined as documented AF, atrial tachycardia, or atrial flutter after the procedure, and the procedure-related complications, such as phrenic nerve palsy (PNP), cardiac tamponade/pericardial effusion, vascular complications, and stroke/asymptomatic cerebral lesions (ACL). Secondary outcomes included procedure and fluoroscopy tines (i.e., duration), and left atrial (LA) dwell time.

### 2.4 Statistical Analysis

Statistical analysis was performed using Stata Release 15.1 (StataCorp LLC, College Station, TX, USA). Outcomes for categorical variables are expressed as odds ratio (OR) with corresponding 95% confidence interval (CI), while continuous variables are expressed as weighted mean difference (WMD) with corresponding 95% CI. A random-effects model was used for all comparisons. Heterogeneity between studies was evaluated using Cochran’s Q test and the $I^{2}$ statistic, with an $I^{2}$ index of >50% indicating high heterogeneity. Possible sources of significant heterogeneity were further investigated through sensitivity and subgroup analyses. Publication bias was evaluated using funnel plots based on primary outcomes; Egger’s and Begg’s tests were used to assess statistical bias. The study protocol was registered at PROSPERO (CRD42023444776).

## 3. Results

### 3.1 Eligible Studies and Characteristics

**Fig. 1.**
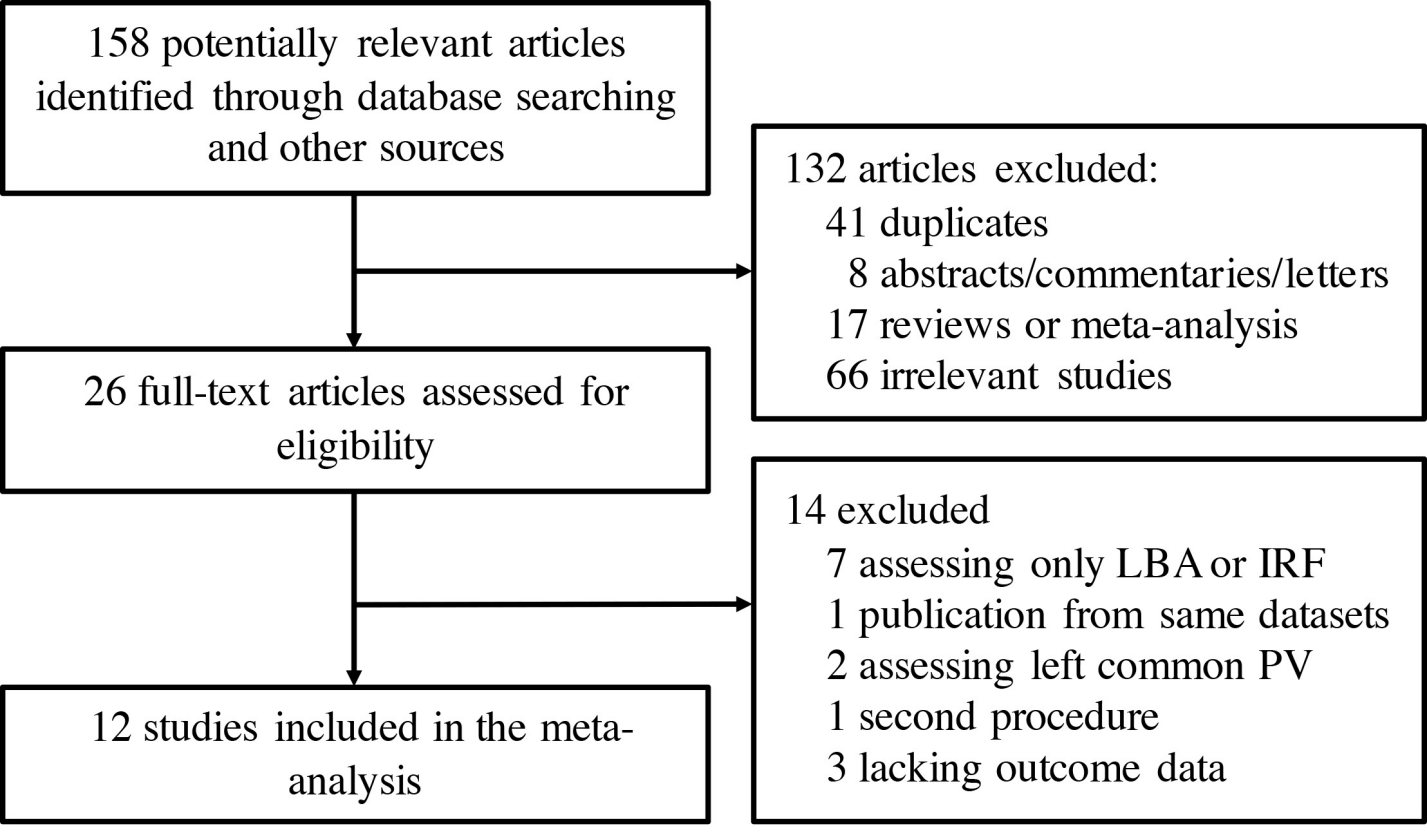
**Flow chart of the systematic literature research. **LBA, laser balloon ablation; IRF, irrigated radiofrequency ablation; PV, pulmonary vein.

Twelve trials [8, 9, 10, 11, 12, 13, 14, 15, 16, 17, 18, 19] were included in this study (Fig. 1). A total of 1274 patients with AF who underwent initial CA treatment were divided into 2 groups and analyzed: LBA (n = 633) versus (vs) RFA (n = 641). Baseline characteristics of the included studies are summarized in Table 1 (Ref. [8, 9, 10, 11, 12, 13, 14, 15, 16, 17, 18, 19]). Five studies [9, 10, 14, 15, 18] were RCTs, whereas the others were prospective or retrospective non-randomized studies. Ten studies [8, 9, 10, 11, 12, 14, 15, 17, 18, 19] used LBA with a first generation laser balloon (LB1), while LB2/3 was performed in 2 studies [13, 16]. RFA-CF was performed in 4 studies [11, 13, 16, 17], whereas RFA without contact-force techniques (RFA-nCF) was performed in 6 studies [8, 10, 12, 14, 18, 19]. Five studies included only patients with paroxysmal atrial fibrillation (PAF) [9, 10, 13, 14, 18], three studies included only patients with persistent atrial fibrillation (PerAF) [8, 15, 17], and a mixed AF population was included in the remaining studies (the percentage of patients with PAF ranged from 22.2% to 86.7%). There were 792 PAF patients and 482 PerAF patients in total. The mean age ranged from 56.8 to 66.5 years, and the mean follow-up duration was 13.3 months. According to evaluation results using the Cochrane Collaboration tool [6] and ROBINS-I tool [7], all included clinical trials were of good quality. Funnel plot analysis demonstrated no significant bias, which was also validated by Begg’s and Egger’s tests (*p* = 0.93 and *p* = 0.91, respectively) (**Supplementary Fig. 1**).

**Table 1.** **Baseline characteristics of the included studies**.

First Author	Year	Study type	N	PAF (%)	LBA Protocol	RFA Protocol	Mean age (years)	Male (%)	Mean LVEF (%)	Mean LAd (mm)	DM (%)	Hypertension (%)	CAD (%)	Follow-up
Seki [16]	2022	Prospective	100	81	LB1	RFA-CF	65.5	77	57.5	38.5	9	47	10	21 m
Skeete [17]	2022	Retrospective	204	0	LB2/LB3	RFA-CF	64.7	59.3	50	45	30	75.5	25.5	24 m
Guenancia [13]	2021	Retrospective	100	100	LB2/LB3	RFA-CF	62	70	60	NR	4	39	10	12 m
Gao [12]	2021	Prospective	27	22.2	LB1	RFA-nCF	64.5	90	58.6	NR	11	67	15	12 m
Üçer [18]	2020	RCT	50	100	LB1	RFA-nCF	62.5	50	60.8	43.1	22	20	26	12 m
Figueras I Ventura [11]	2018	Prospective	30	86.7	LB1	RFA-CF	58	73.3	NR	41	NR	NR	NR	12 m
Schmidt [15]	2017	RCT	134	0	LB1	RFA-CF/nCF	66	63	61	43	9.7	72.4	18.7	12 m
Bordignon [8]	2016	Prospective	80	0	LB1	RFA-nCF	66.5	71.3	60.5	42.5	11.3	85	17.5	365 d
Dukkipati [10]	2015	RCT	353	100	LB1	RFA-nCF	59.9	66.4	60.4	40	12.6	58.8	20.8	12 m
Casella [9]	2014	RCT	75	100	LB1	RFA-CF/nCF	56.8	81.3	62	42.4	NR	34.7	NR	12 m
Wissner [19]	2014	Prospective	66	69.7	LB1	RFA-nCF	63.3	60.6	64.3	43	10.6	65.2	6.1	6 m
Schmidt [14]	2013	RCT	66	100	LB1	RFA-nCF	64	NR	59.5	40.5	6.1	72.7	15.2	12 m

PAF, paroxysmal atrial fibrillation; LBA, laser balloon ablation; RFA, radiofrequency ablation; LB1, LBA with first-generation laser balloon; LB2, LBA with second-generation laser balloon; LB3, LBA with third-generation laser balloon; RFA-CF, RFA with contact-force technology; RFA-nCF, RFA without contact force technology; LVEF, left ventricular ejection fraction; LAd, left atrial diameter; DM, diabetes mellitus; CAD, coronary artery disease; NR, not reported; RCT, randomized controlled trial; N, number; m, month; d, day.

### 3.2 Primary Endpoints

#### 3.2.1 Freedom from ATA

**Fig. 2.**
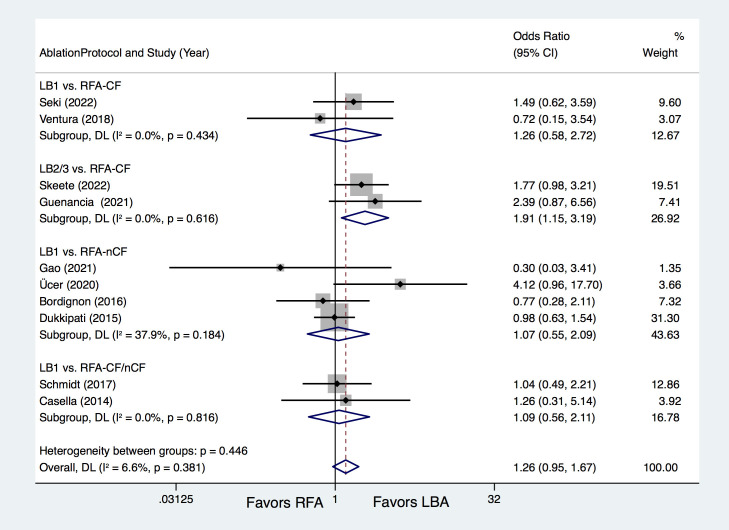
**Forest plots for the outcome of freedom from atrial tachyarrhythmia (ATA) according to the ablation protocols.** LBA, laser balloon ablation; RFA, irrigated radiofrequency ablation; LB1, LBA with first-generation laser balloon; LB2/3, LBA with second- and third-generation laser balloon; RFA-CF, RFA with contact-force technology; RFA-nCF, RFA without contact-force technology; DL, DerSimonian and Laird random-effects model.

Of the included trials, 10 studies [8, 9, 10, 11, 12, 13, 15, 16, 17, 18] provided information regarding outcomes for freedom from ATA, with results indicating that LBA and RFA yielded similar rates (72.5% vs. 68.7%; OR = 1.26, 95% CI 0.95–1.67, *p* = 0.111), and no significant heterogeneity ($I^{2}$ = 6.6%) (**Supplementary Fig. 2**). Additional subgroup analyses were also performed and revealed that patients treated using LB2/3 experienced significantly higher rates of freedom from ATA, compared with RFA-CF (77.8% vs. 64.9%; OR = 1.91, 95% CI 1.15–3.19, *p* = 0.013) (Fig. 2). No such differences were found between LB1 vs. RFA-CF (OR = 1.26, *p* = 0.560), LB1 vs. RFA-nCF (OR = 1.07, *p* = 0.846) and LB1 vs. RFA-CF/nCF (OR = 1.09, *p* = 0.805) (Fig. 2). In addition, LBA and RFA yielded comparable rates of freedom from ATA in the PAF (OR = 1.56, *p* = 0.176), PerAF (OR = 1.26, *p* = 0.336), and mixed AF (OR = 1.10, *p* = 0.794) populations (**Supplementary Fig. 3**).

**Fig. 3.**
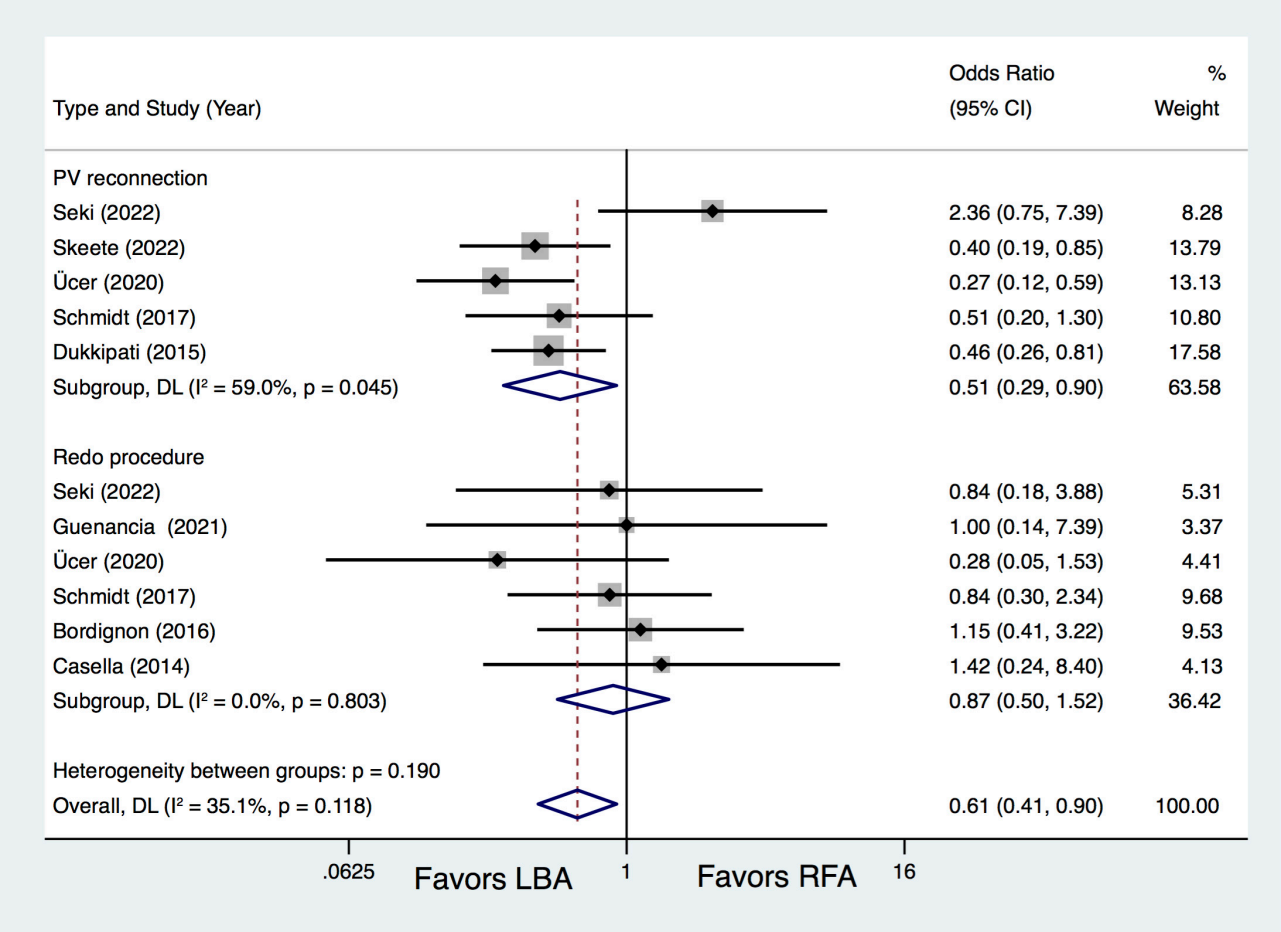
**Forest plots for the outcome of PV reconnection and redo procedure.** LBA, laser balloon ablation; RFA, irrigated radiofrequency ablation; PV, pulmonary vein; DL, DerSimonian and Laird random-effects model.

Five studies [10, 15, 16, 17, 18] provided additional data regarding PV reconnection and redo procedure rates. Results of analysis revealed that LBA resulted in remarkably fewer PV reconnections than RFA (OR = 0.51, 95% CI 0.29–0.90, *p *= 0.021), with no statistical difference regarding to redo procedure rates (OR = 0.87, 95% CI 0.50–1.52, *p* = 0.634) (Fig. 3).

#### 3.2.2 Procedure Related Complications

**Fig. 4.**
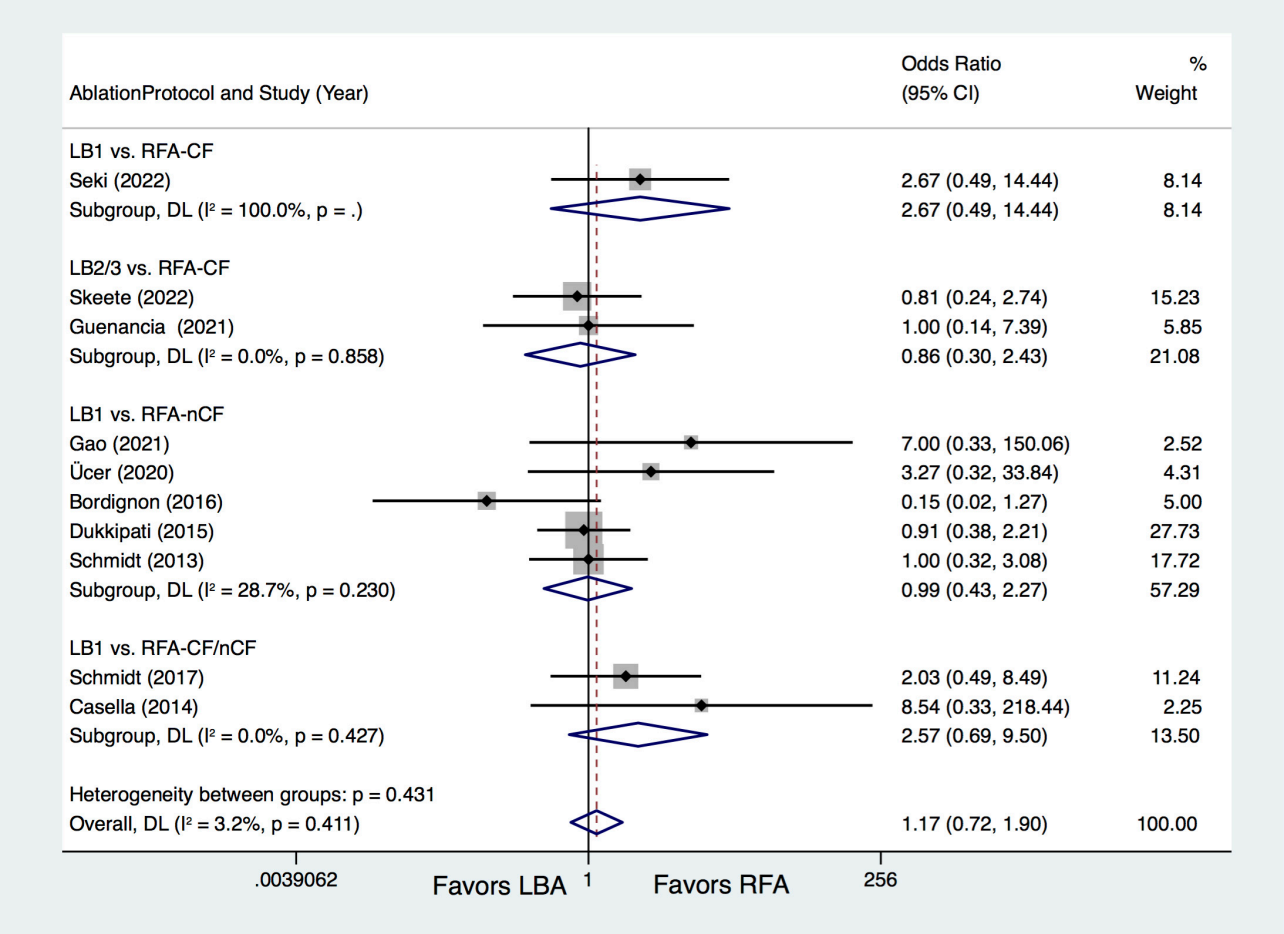
**Forest plots for total procedure-related complications according to the ablation protocols.** LBA, laser balloon ablation; RFA, irrigated radiofrequency ablation; LB1, LBA with first-generation laser balloon; LB2/3, LBA with second- and third-generation laser balloon; RFA-CF, RFA with contact-force technology; RFA-nCF, RFA without contact-force technology; DL, DerSimonian and Laird random-effects model.

**Fig. 5.**
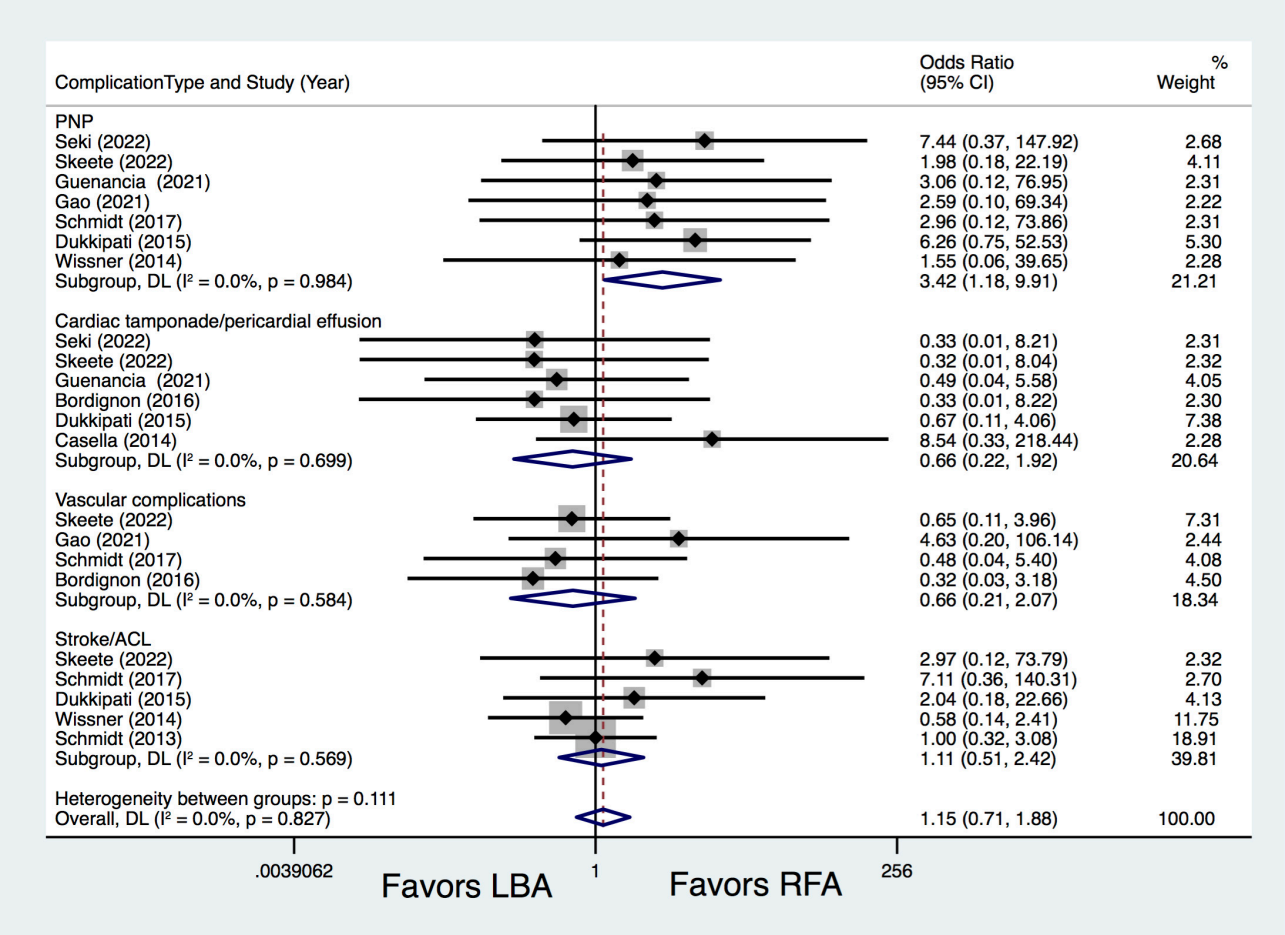
**Forest plots for different procedure-related complications. **PNP, phrenic nerve palsy; ACL, asymptomatic cerebral lesions; LBA, laser balloon ablation; RFA, irrigated radiofrequency ablation; DL, DerSimonian and Laird random-effects model.

Ten studies [8, 9, 10, 12, 13, 14, 15, 16, 17, 18] compared procedure-related complications, with similar complications rates between LBA and RFA (7.7% vs. 6.5%; OR = 1.17, 95% CI 0.72–1.90, *p* = 0.536). Further subgroup analyses also revealed comparable total complications rates between LB1 vs. RFA-CF (OR = 2.67, *p* = 0.255), LB2/3 vs. RFA-CF (OR = 0.86, *p* = 0.770), LB1 vs. RFA-nCF (OR = 0.99, *p* = 0.975) and LB1 vs. RFA-CF/nCF (OR = 2.57, *p *= 0.158) (Fig. 4). Subgroup analysis of complication types revealed that LBA resulted in significantly higher PNP rates than RFA (2.8% vs. 0.4%; OR = 3.42, 95% CI 1.18–9.91, *p* = 0.023) (Fig. 5). The occurrences of cardiac tamponade/pericardial effusion (OR = 0.66, *p *= 0.442), vascular complications (OR = 0.66, *p* = 0.475), and stroke/ACL (OR = 1.11, *p* = 0.786) were comparable between the LBA and RFA groups (Fig. 5).

### 3.3 Secondary Endpoints

**Fig. 6.**
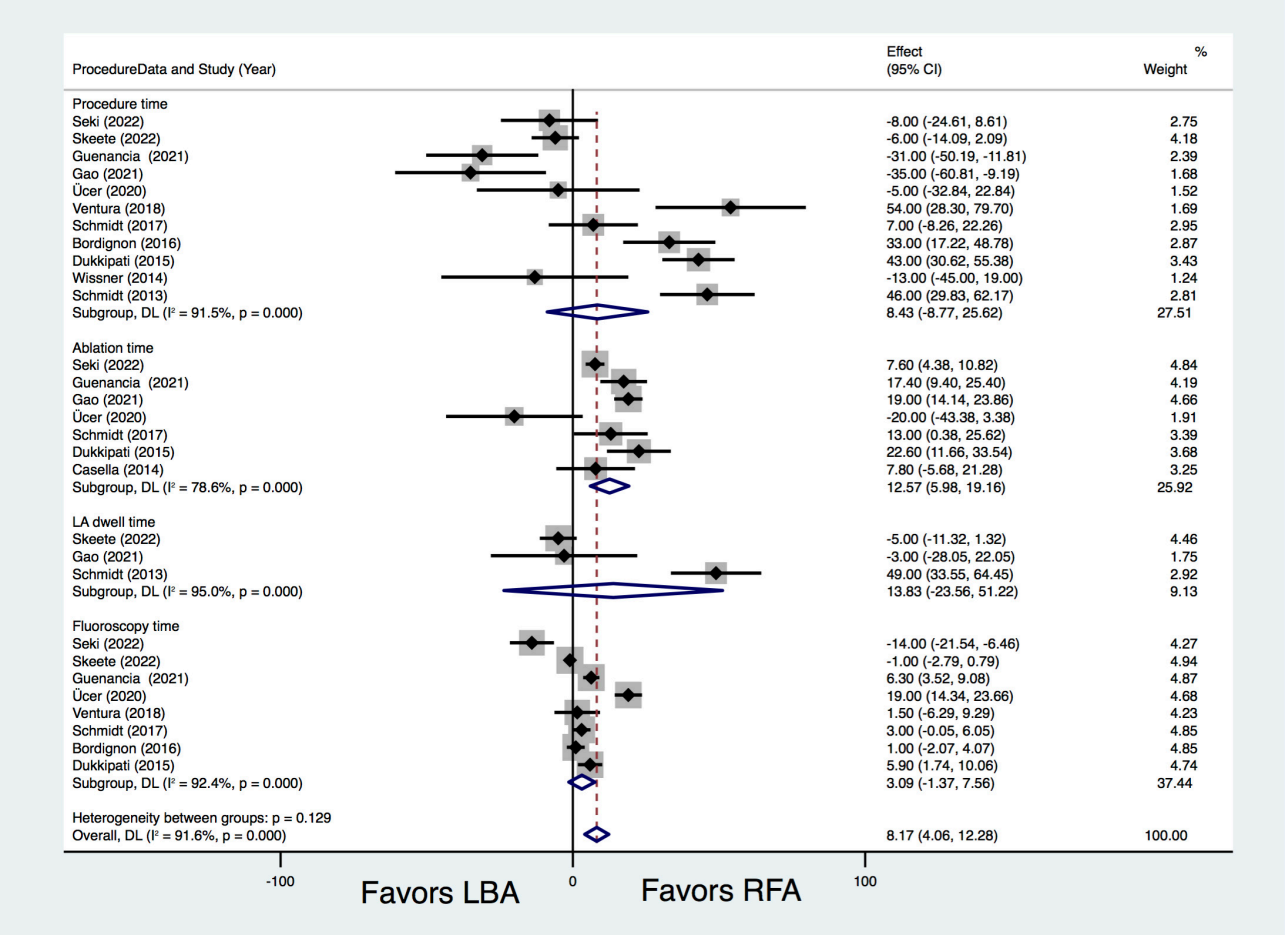
**Forest plots for the outcome of procedure characteristics.** LBA, laser balloon ablation; RFA, irrigated radiofrequency ablation; LA, left atrium; DL, DerSimonian and Laird random-effects model.

Ablation time was found to be significantly longer in the LBA therapy group than that in the RFA therapy group (WMD = 12.57 min; 95% CI 5.98–19.16 min, *p* = 0.00). Whereas, procedure time (WMD = 8.43 min, *p* = 0.337), fluoroscopy time (WMD = 3.09 min, *p *= 0.174) and LA dwell time (WMD = 13.83 min, *p* = 0.468) were comparable between LBA and RFA. There was considerable heterogeneity in the comparisons ($I^{2}$ varied from 78.6% to 95.0%) (Fig. 6). Further subgroup analyses revealed comparable procedure times for LB1 vs. RFA-CF (WMD = 22.19 min, *p *= 0.474), LB2/3 vs. RFA-CF (WMD = –16.92 min, *p* = 0.172), LB1 vs. RFA-nCF (WMD = 13.91 min, p = 0.253) and LB1 vs. RFA-CF/nCF (WMD = 7.00 min, *p* = 0.369) (**Supplementary Fig. 4**). Similar results were found regarding fluoroscopy duration in comparison to LB1 vs. RFA-CF (WMD = –6.82 min, *p* = 0.418), LB2/3 vs. RFA-CF (WMD = 2.57 min, *p* = 0.481), LB1 vs. RFA-nCF (WMD = 8.52 min, *p* = 0.099) and LB1 vs. RFA-CF/nCF (WMD = 3.00 min, *p* = 0.054) (**Supplementary Fig. 5**). However, LBA treatment required a significantly longer fluoroscopy time than RFA for patients with PAF (WMD = 10.25 min; 95% CI 2.72–17.78 min, *p* = 0.008) (**Supplementary Fig. 6**). No such differences were observed for patients with PerAF (WMD = 0.75 min, *p* = 0.546) or those with mixed AF (WMD = –6.28 min, *p* = 0.418) (**Supplementary Fig. 6**).

## 4. Discussion

To the best of our knowledge, this is the first systematic review and meta-analysis to compare the safety, efficacy, and procedural characteristics of LBA and RFA. The main findings are described as follows. First, LBA and RFA yielded comparable rates of freedom from ATA (72.5% vs. 68.7%), and LB2/3 yielded significantly higher freedom from ATA rates than RFA-CF (77.8% vs. 64.9%); Second, LBA demonstrated significantly lower PV reconnection rates than RFA. Third, LBA and RFA exhibited similar safety profiles. Higher PNP rates were observed in the LBA group. LBA required a longer fluoroscopy time than RFA for patients with PAF, as well as a longer ablation time.

CA therapy has been applied and recommended to restore and maintain sinus rhythm in symptomatic patients with AF, with PVI being the cornerstone strategy [4]. Irrigated RFA treatment is a very well-established technique for achieving PVI. Although many technical innovations, such as the contact-force technique, have been proposed, RFA continues to be technically intricate, and time-consuming, and requires a long learning curve [3]. To simplify the conventional RFA procedure, many balloon-based CA technologies have been proposed in recent years and have yielded efficacy and safety outcomes similar to those of RFA [4, 10].

Among the balloon-based techniques, LBA is a really unique method that provides direct and real-time visualization of the tissues from an endoscopic view [10]. In addition, LBA can adapt to various PV anatomies with a compliant balloon, which can also provide operators with greater flexibility with energy titration [17]. Since the first multicenter RCT by Dukkipati *et al*. [10], many trials have compared the safety and efficacy of LBA vs. RFA, although the results remain controversial, especially when the newer generations of both techniques were introduced [13].

In the present study, LBA yielded comparable rates of freedom from ATA compared with RFA. This is consistent with the results of large-scale RCTs of other balloon-based techniques, such as cryoballoon ablation (CBA) [4], demonstrating the non-inferiority of these balloon devices to RFA. It should be noted that each technique has evolved and has been widely used in recent years. However, only 2 of the 12 studies used LB2/LB3, and only 4 studies used RFA-CF exclusively. Additional subgroup analysis revealed that LB2/3 yielded higher freedom from ATA rates than RFA-CF (77.8% vs. 64.9%). This may be reasonable, because the improvements between LB1 and LB2/3 were significant. LB2 has a more compliant balloon than LB1 and has much better catheter stability during lesion delivery [17]. The LB3 catheter has added to the technical innovation of continuous circular energy delivery, which has resulted in continuous lesions, fewer gaps, and a higher PVI durability [20]. A pivotal study investigating LB3 reported significantly higher acute PVI rates and better clinical efficacy outcomes compared with that of LB1 [21]. Thus, use of the newest generations of LB2/3 may yield better clinical outcomes, even when compared with RFA-CF.

Another important advantage of LBA over RFA for the primary endpoint may be the significantly lower PV reconnection rates observed in the LBA group. It has been reported that 80% of patients with AF who experienced a recurrence exhibit at least 1 reconnected PV [22]. The long-term efficacy of CA is limited mainly by PV reconnection(s) [23]. Although it may be technically challenging for point-by-point RFA to achieve a contiguous necrotic loop surrounding the PVs [10], LBA may have several advantages in delivering overlapping contiguous lesions through direct tissue visualization, which ensures the wide-area circumferential ablation and fewer PV gaps [17, 18]. As was reported by Dukkipati *et al*. [24], 86%–90% of the PVs remained isolated 3 months after the LBA treatment. In contrast, the disconnection rate was only 57% after the PVI with RFA [25]. However, it should be noted that, only 2 studies, including 304 participants, directly compared LB2/3 with RFA-CF, and both patients with PAF and PerAF were included. Furthermore, only Holter electrocardiography was used as monitoring methods in both studies, rather than continuous rhythm monitoring, which may inevitably cause certain bias when evaluating the outcomes such as AF recurrences. Thus, this result should be interpreted with caution, and more clinical evidence from comparisons of LB2/3 and RFA-CF is needed.

Regarding the safety profile, there was no significant difference in procedure-related complications between LBA and RFA (7.7% vs. 6.5%). The rates were similar to those of previous studies, which reported a prevalence of 0.8%–16.3% [26]. A previous study reviewed various complications of the CA procedure in 3000 patients with AF [27], and reported that the occurrence of cardiac tamponade was 1.1% in those receiving RFA, while it was only 0.1% for balloon catheters (LBA and CBA). However, our study found similar cardiac tamponade rates in the LBA and CBA groups. The procedure-related complications rates between the different generations of LBA and RFA yielded similar results, demonstrating their relatively similar safety profiles. Among the complications, PNP rates were higher in the LBA group. PNP is a typical complication of balloon-based ablation; however, it resolves in most patients during follow-up [28]. The PNP rate in this study was also consistent with that in a previously published, single-arm, meta-analysis of LBA in patients with AF [29]. It has also been reported that patients treated with LB3 experienced no PNP events, possibly due to its more compliant balloon [21]. Thus, the safety outcomes of PNP may be greatly improved with the widespread use of LB2 and LB3.

The introduction of balloon-based technologies helps to reduce the procedural complexity of RFA. Previous studies have demonstrated that second-generation CBA requires a shorter procedure time than RFA-CF [3]. In the present meta-analysis, the total fluoroscopy and procedure times were similar for LBA and RFA were similar; however, more ablation time was required for LBA. In contrast to CBA, LBA lacks the ability to record the real-time PV potentials, which requires PVI validation and may cause increased procedure and ablation times when the first-round PVI fails [30]. Additionally, owing to its visually-guided features, LBA requires less fluoroscopy time. However, the total fluoroscopy time was comparable with that of RFA and was even longer in the subgroup of patients with PAF. A possible explanation may be that the majority studies applied LB1, while a plenty of evidence has supported greatly shortened procedure and fluoroscopy times with LB2/LB3, attributed the “RAPID” mode [21]. Significant heterogeneity and learning-curve effects should also be considered. Continued improvements in increased PVI rates, as well as reduced procedure time were observed along with the increased operator experience for LBA [31]. On the other hand, the new techniques introduced for RFA, such as the contact-force technique, the high-power and short-duration strategy, and the application of ablation index-guided ablation, could also greatly contribute to improving the efficacy and reducing the procedure duration [32]. Therefore, large-scale RCTs investigating the efficacy and safety profiles of LBA and CBA are warranted.

## 5. Study Limitations

The present study had several limitations, the first of which was the relatively small sample size, and the evidence was mostly from non-randomized clinical trials, because only 5 of the studies were RCTs. However, all the included studies were evaluated to be of good quality. Second, patients with mixed AF types were enrolled in the study. Additional subgroup analyses were thus performed based on the different clinical AF types and procedural protocols. Third, the ATA recurrence monitoring protocols and the endpoint definitions regarding to procedure-related complications were not uniform, which may have resulted in potential bias. In addition, LB1 was applied in most studies (10 of the 12 trials), while the improvements between LB1and LB2/3 were large. The present results should be further updated for LBA with the newest-generations technology. Finally, some heterogeneities were found when analyzing the results of the secondary outcomes, and we performed additional subgroup analyses; however, these results need to be interpreted with caution.

## 6. Conclusions

LBA and RFA in patients with AF yielded comparable safety and efficacy in terms of the rates of freedom from ATA and procedure-related complications. LB2/3 had significantly higher rates of freedom from ATA than RFA-CF, whereas, LBA was associated with a higher risk for PNP. Compared with RFA, LBA was found to have significantly lower PV reconnection rates but required significantly longer ablation and fluoroscopy times for patients with PAF. Large-scale RCTs comparing LBA and RFA are warranted to validate the findings of the current study and inform the latest recommendations.

## Data Availability

The datasets used during the current study are available from the corresponding author on reasonable request.

## Supplementary Material

Supplementary material associated with this article can be found, in the online version, at https://doi.org/10.31083/j.rcm2506205.

## References

[1] Schnabel RB, Yin X, Gona P, Larson MG, Beiser AS, McManus DD (2015). 50 year trends in atrial fibrillation prevalence, incidence, risk factors, and mortality in the Framingham Heart Study: a cohort study. *Lancet (London, England)*.

[2] Hindricks G, Potpara T, Dagres N, Arbelo E, Bax JJ, Blomström-Lundqvist C (2021). 2020 ESC Guidelines for the diagnosis and management of atrial fibrillation developed in collaboration with the European Association for Cardio-Thoracic Surgery (EACTS): The Task Force for the diagnosis and management of atrial fibrillation of the European Society of Cardiology (ESC) Developed with the special contribution of the European Heart Rhythm Association (EHRA) of the ESC. *European Heart Journal*.

[3] Wu C, Li X, Lv Z, Chen Q, Lou Y, Mao W (2021). Second-generation cryoballoon versus contact force radiofrequency ablation for atrial fibrillation: an updated meta-analysis of evidence from randomized controlled trials. *Scientific Reports*.

[4] Kuck KH, Brugada J, Fürnkranz A, Metzner A, Ouyang F, Chun KRJ (2016). Cryoballoon or Radiofrequency Ablation for Paroxysmal Atrial Fibrillation. *The New England Journal of Medicine*.

[5] Heeger CH, Tiemeyer CM, Phan HL, Meyer-Saraei R, Fink T, Sciacca V (2020). Rapid pulmonary vein isolation utilizing the third-generation laserballoon - The PhoeniX registry. *International Journal of Cardiology. Heart & Vasculature*.

[6] Higgins JPT, Altman DG, Gøtzsche PC, Jüni P, Moher D, Oxman AD (2011). The Cochrane Collaboration’s tool for assessing risk of bias in randomised trials. *BMJ (Clinical Research Ed.)*.

[7] Sterne JA, Hernán MA, Reeves BC, Savović J, Berkman ND, Viswanathan M (2016). ROBINS-I: a tool for assessing risk of bias in non-randomised studies of interventions. *BMJ (Clinical Research Ed.)*.

[8] Bordignon S, Boehmer MC, Klostermann A, Fuernkranz A, Perrotta L, Dugo D (2016). Visually guided pulmonary vein isolation in patients with persistent atrial fibrillation. *Europace: European Pacing, Arrhythmias, and Cardiac Electrophysiology: Journal of the Working Groups on Cardiac Pacing, Arrhythmias, and Cardiac Cellular Electrophysiology of the European Society of Cardiology*.

[9] Casella M, Dello Russo A, Russo E, Al-Mohani G, Santangeli P, Riva S (2014). Biomarkers of myocardial injury with different energy sources for atrial fibrillation catheter ablation. *Cardiology Journal*.

[10] Dukkipati SR, Cuoco F, Kutinsky I, Aryana A, Bahnson TD, Lakkireddy D (2015). Pulmonary Vein Isolation Using the Visually Guided Laser Balloon: A Prospective, Multicenter, and Randomized Comparison to Standard Radiofrequency Ablation. *Journal of the American College of Cardiology*.

[11] Figueras I Ventura RM, Mǎrgulescu AD, Benito EM, Alarcón F, Enomoto N, Prat-Gonzalez S (2018). Postprocedural LGE-CMR comparison of laser and radiofrequency ablation lesions after pulmonary vein isolation. *Journal of Cardiovascular Electrophysiology*.

[12] Gao X, Chang D, Bilchick KC, Hussain SK, Petru J, Skoda J (2021). Left atrial thickness and acute thermal injury in patients undergoing ablation for atrial fibrillation: Laser versus radiofrequency energies. *Journal of Cardiovascular Electrophysiology*.

[13] Guenancia C, Hammache N, Docq C, Benali K, Hooks D, Echivard M (2021). Efficacy and Safety of Second and Third-Generation Laser Balloon for Paroxysmal Atrial Fibrillation Ablation Compared to Radiofrequency Ablation: A Matched-Cohort. *Journal of Cardiovascular Development and Disease*.

[14] Schmidt B, Gunawardene M, Krieg D, Bordignon S, Fürnkranz A, Kulikoglu M (2013). A prospective randomized single-center study on the risk of asymptomatic cerebral lesions comparing irrigated radiofrequency current ablation with the cryoballoon and the laser balloon. *Journal of Cardiovascular Electrophysiology*.

[15] Schmidt B, Neuzil P, Luik A, Osca Asensi J, Schrickel JW, Deneke T (2017). Laser Balloon or Wide-Area Circumferential Irrigated Radiofrequency Ablation for Persistent Atrial Fibrillation: A Multicenter Prospective Randomized Study. *Circulation. Arrhythmia and Electrophysiology*.

[16] Seki R, Nagase T, Asano S, Fukunaga H, Inoue K, Sekiguchi Y (2022). Radiofrequency Current Versus Balloon-Based Ablation for Atrial Fibrillation. *The American Journal of Cardiology*.

[17] Skeete J, Sharma PS, Kenigsberg D, Pietrasik G, Osman AF, Ravi V (2022). Wide area circumferential ablation for pulmonary vein isolation using radiofrequency versus laser balloon ablation. *Journal of Arrhythmia*.

[18] Üçer E, Fredersdorf S, Seegers J, Poschenrieder F, Hauck C, Maier L (2020). High Predictive Value of Adenosine Provocation in Predicting Atrial Fibrillation Recurrence After Pulmonary Vein Isolation With Visually Guided Laser Balloon Compared With Radiofrequency Ablation. *Circulation Journal: Official Journal of the Japanese Circulation Society*.

[19] Wissner E, Metzner A, Neuzil P, Petru J, Skoda J, Sediva L (2014). Asymptomatic brain lesions following laserballoon-based pulmonary vein isolation. *Europace: European Pacing, Arrhythmias, and Cardiac Electrophysiology: Journal of the Working Groups on Cardiac Pacing, Arrhythmias, and Cardiac Cellular Electrophysiology of the European Society of Cardiology*.

[20] Tohoku S, Bordignon S, Chen S, Bologna F, Urbanek L, Operhalski F (2021). Validation of lesion durability following pulmonary vein isolation using the new third-generation laser balloon catheter in patients with recurrent atrial fibrillation. *Journal of Cardiology*.

[21] Schmidt B, Petru J, Chun KRJ, Sediva L, Bordignon S, Chen S (2021). Pivotal Study of a Novel Motor-Driven Endoscopic Ablation System. *Circulation. Arrhythmia and Electrophysiology*.

[22] Medi C, Sparks PB, Morton JB, Kistler PM, Halloran K, Rosso R (2011). Pulmonary vein antral isolation for paroxysmal atrial fibrillation: results from long-term follow-up. *Journal of Cardiovascular Electrophysiology*.

[23] Ouyang F, Tilz R, Chun J, Schmidt B, Wissner E, Zerm T (2010). Long-term results of catheter ablation in paroxysmal atrial fibrillation: lessons from a 5-year follow-up. *Circulation*.

[24] Dukkipati SR, Neuzil P, Skoda J, Petru J, d’Avila A, Doshi SK (2010). Visual balloon-guided point-by-point ablation: reliable, reproducible, and persistent pulmonary vein isolation. *Circulation. Arrhythmia and Electrophysiology*.

[25] Willems S, Steven D, Servatius H, Hoffmann BA, Drewitz I, Müllerleile K (2010). Persistence of pulmonary vein isolation after robotic remote-navigated ablation for atrial fibrillation and its relation to clinical outcome. *Journal of Cardiovascular Electrophysiology*.

[26] De Greef Y, Ströker E, Schwagten B, Kupics K, De Cocker J, Chierchia GB (2018). Complications of pulmonary vein isolation in atrial fibrillation: predictors and comparison between four different ablation techniques: Results from the MIddelheim PVI-registry. *Europace: European Pacing, Arrhythmias, and Cardiac Electrophysiology: Journal of the Working Groups on Cardiac Pacing, Arrhythmias, and Cardiac Cellular Electrophysiology of the European Society of Cardiology*.

[27] Chun KRJ, Perrotta L, Bordignon S, Khalil J, Dugo D, Konstantinou A (2017). Complications in Catheter Ablation of Atrial Fibrillation in 3,000 Consecutive Procedures: Balloon Versus Radiofrequency Current Ablation. *JACC. Clinical Electrophysiology*.

[28] Okumura Y, Henz BD, Bunch TJ, Dalegrave C, Johnson SB, Packer DL (2009). Distortion of right superior pulmonary vein anatomy by balloon catheters as a contributor to phrenic nerve injury. *Journal of Cardiovascular Electrophysiology*.

[29] Reynolds MR, Zheng Q, Doros G (2018). Laser balloon ablation for AF: A systematic review and meta-analysis. *Journal of Cardiovascular Electrophysiology*.

[30] Chun JKR, Bordignon S, Last J, Mayer L, Tohoku S, Zanchi S (2021). Cryoballoon Versus Laserballoon: Insights From the First Prospective Randomized Balloon Trial in Catheter Ablation of Atrial Fibrillation. *Circulation. Arrhythmia and Electrophysiology*.

[31] Dukkipati SR, Kuck KH, Neuzil P, Woollett I, Kautzner J, McElderry HT (2013). Pulmonary vein isolation using a visually guided laser balloon catheter: the first 200-patient multicenter clinical experience. *Circulation. Arrhythmia and Electrophysiology*.

[32] Castrejón-Castrejón S, Martínez Cossiani M, Ortega Molina M, Escobar C, Froilán Torres C, Gonzalo Bada N (2020). Feasibility and safety of pulmonary vein isolation by high-power short-duration radiofrequency application: short-term results of the POWER-FAST PILOT study. *Journal of Interventional Cardiac Electrophysiology: an International Journal of Arrhythmias and Pacing*.

