# Supplementary material for: Efficacy and Safety of Laser Balloon Versus Irrigated Radiofrequency Ablation as Initial Therapies for Atrial Fibrillation: A Meta-Analysis
Source: Rev Cardiovasc Med. 2024 Jun 3;25(6):205. doi: 10.31083/j.rcm2506205 (PMC11270124; doi:10.31083/j.rcm2506205)
Supplement: Supplementary file 1 [file 2153-8174-25-6-205-s1.zip › Supplementary.pdf]

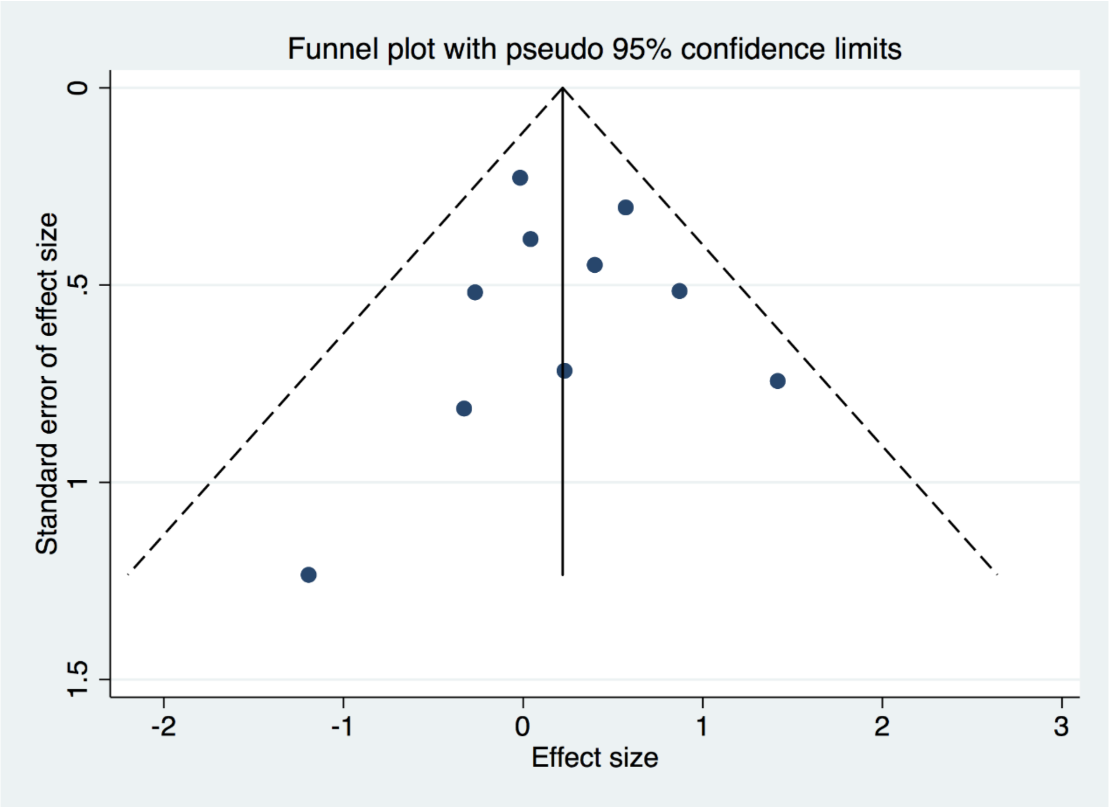

Supplementary Fig.1

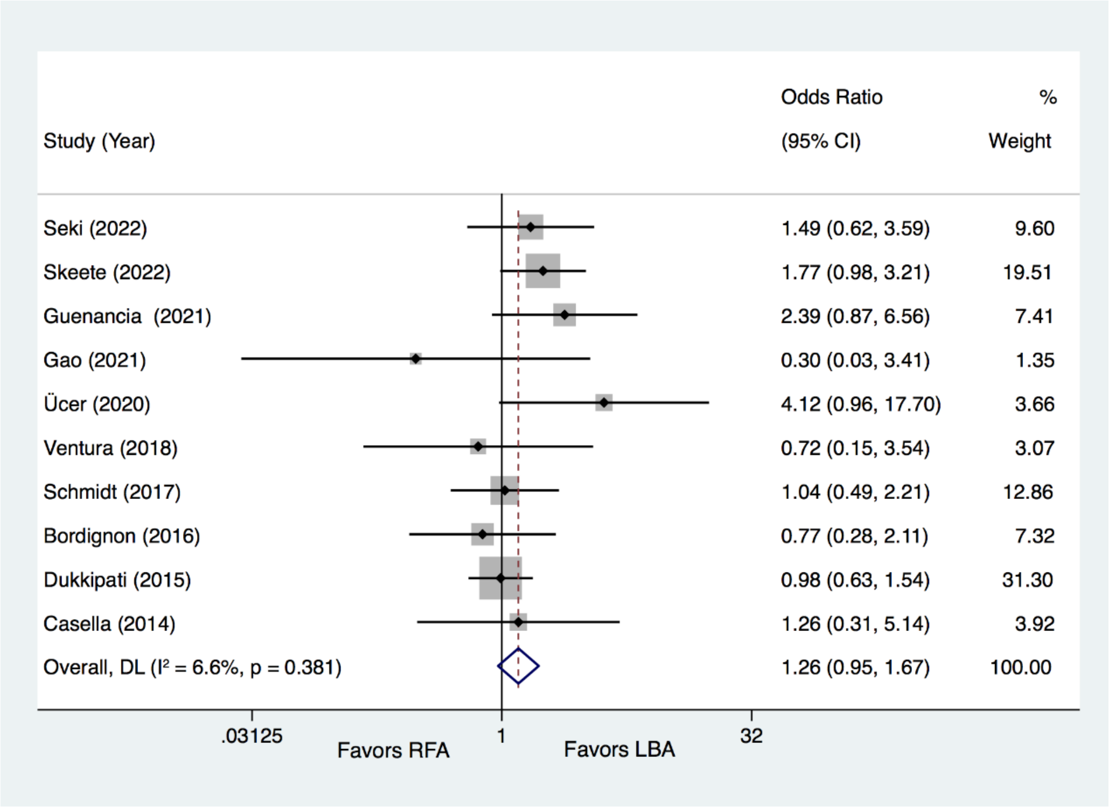

Supplementary Fig.2

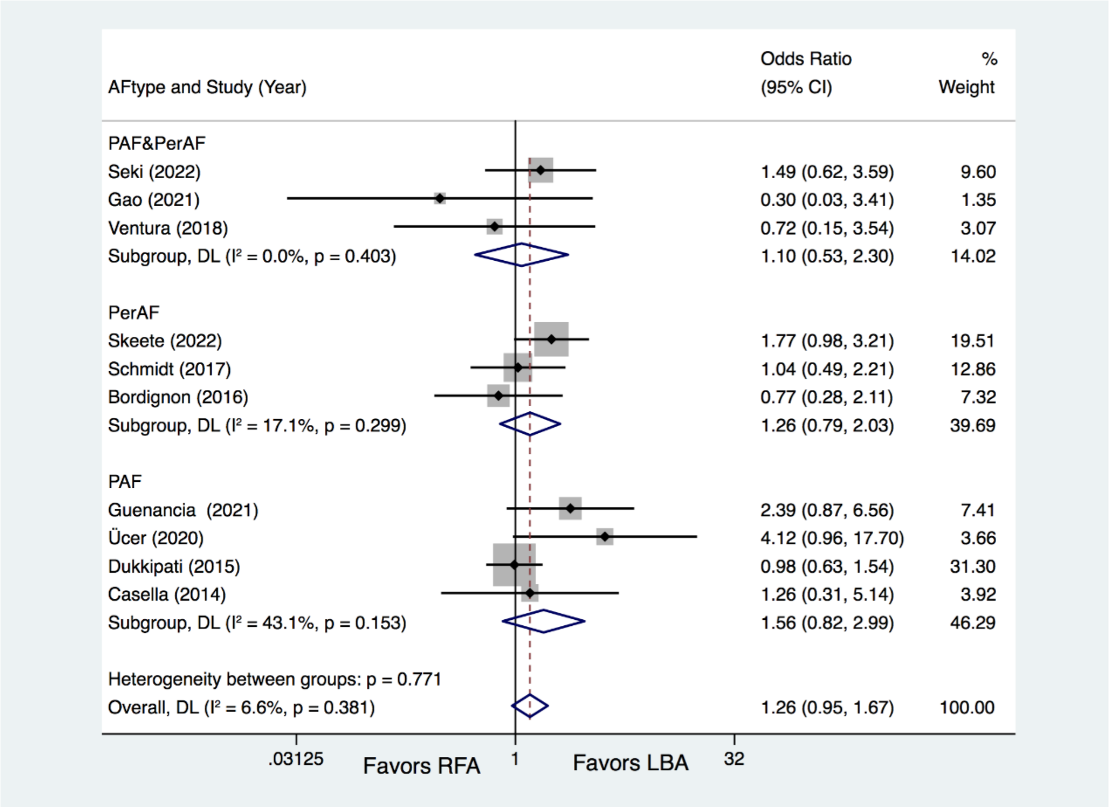

Supplementary Fig.3

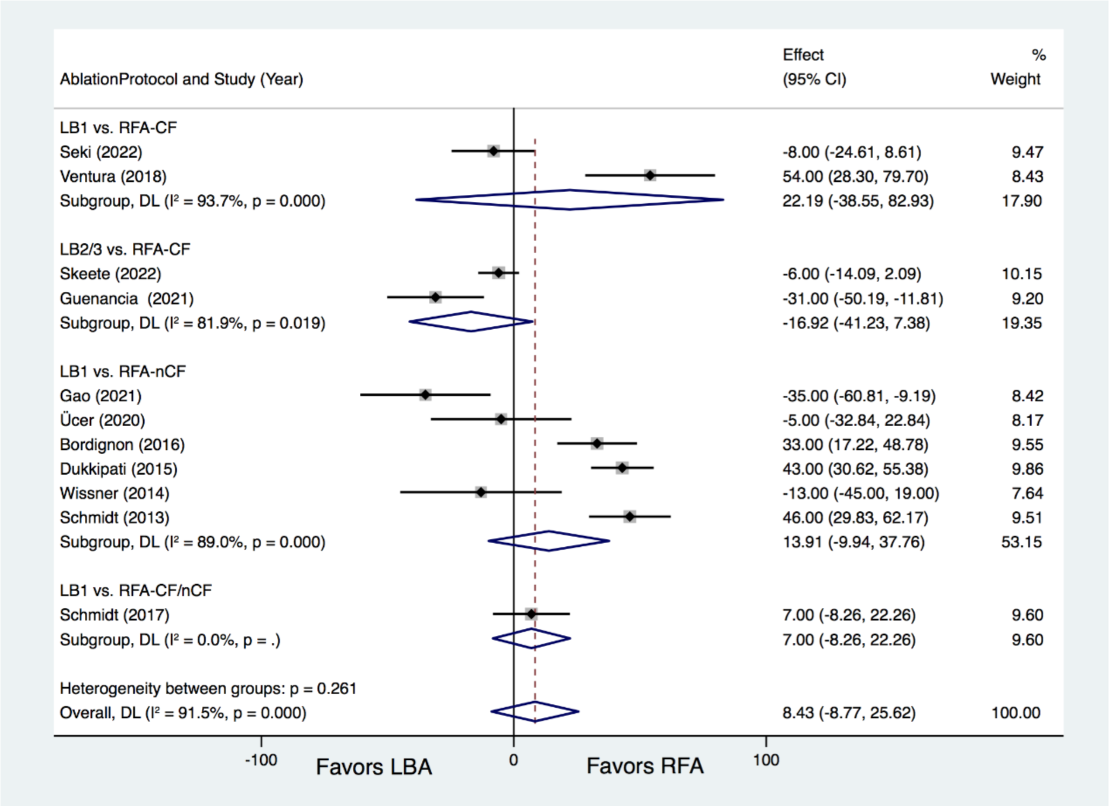

Supplementary Fig.4

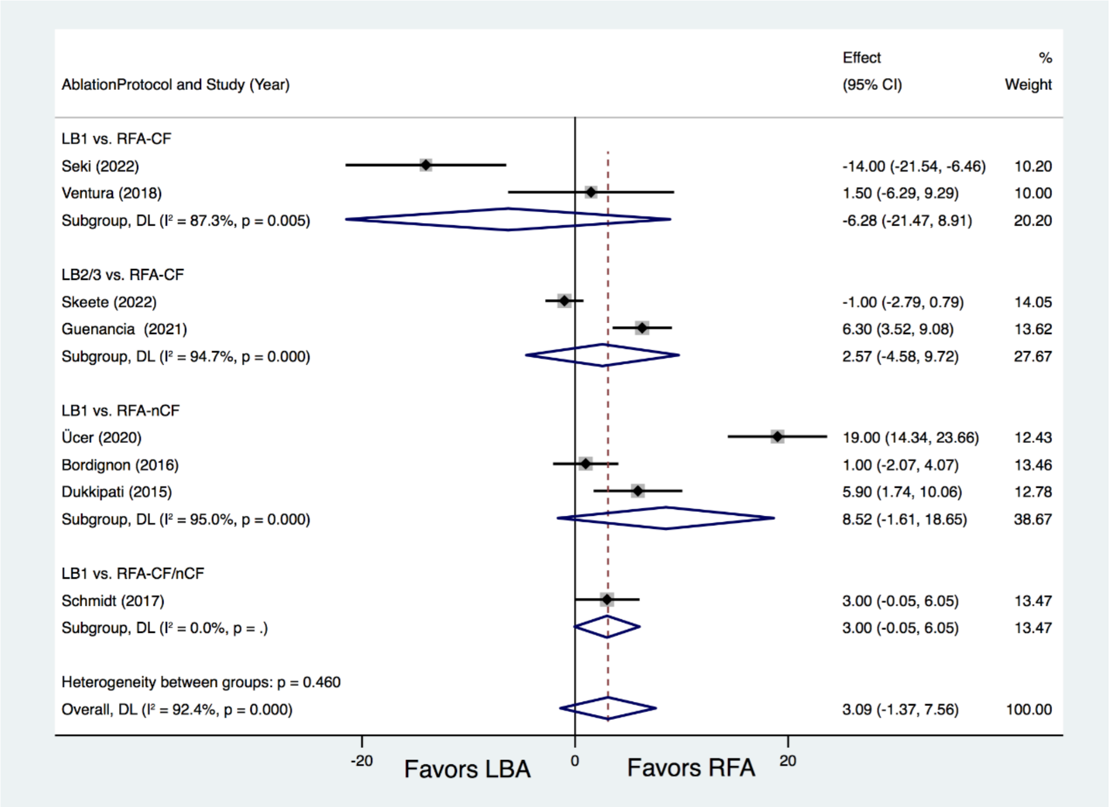

Supplementary Fig.5

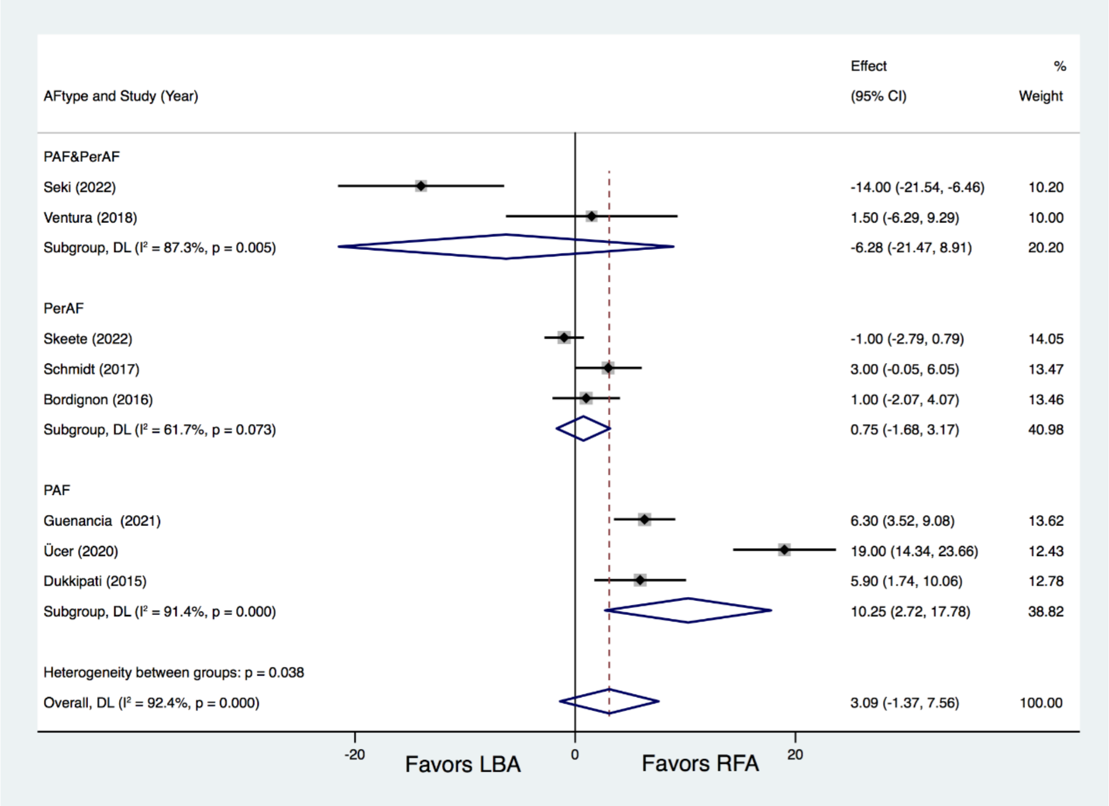

Supplementary Fig.6
